# Ascidian Mitogenomics: Comparison of Evolutionary Rates in Closely Related Taxa Provides Evidence of Ongoing Speciation Events

**DOI:** 10.1093/gbe/evu041

**Published:** 2014-02-25

**Authors:** Francesca Griggio, Ayelet Voskoboynik, Fabio Iannelli, Fabienne Justy, Marie-Ka Tilak, Turon Xavier, Graziano Pesole, Emmanuel J.P. Douzery, Francesco Mastrototaro, Carmela Gissi

**Affiliations:** ^1^Dipartimento di Bioscienze, Università degli Studi di Milano, Milano, Italy; ^2^Institute for Stem Cell Biology and Regenerative Medicine, Stanford University School of Medicine; ^3^Department of Developmental Biology, Stanford University; ^4^Institut des Sciences de l'Evolution de Montpellier (ISEM), Université Montpellier II, France; ^5^Center for Advanced Studies of Blanes (CEAB-CSIC), CSIC, Blanes, Spain; ^6^Istituto di Biomembrane e Bioenergetica, CNR, Bari, Italy; ^7^Dipartimento di Bioscienze, Biotecnologie e Biofarmaceutica, Università degli Studi di Bari, Italy; ^8^Dipartimento di Biologia, Università degli Studi di Bari, Italy

**Keywords:** ascidian, mitochondrial genome, evolutionary rate, species identification

## Abstract

Ascidians are a fascinating group of filter-feeding marine chordates characterized by rapid evolution of both sequences and structure of their nuclear and mitochondrial genomes. Moreover, they include several model organisms used to investigate complex biological processes in chordates. To study the evolutionary dynamics of ascidians at short phylogenetic distances, we sequenced 13 new mitogenomes and analyzed them, together with 15 other available mitogenomes, using a novel approach involving detailed whole-mitogenome comparisons of conspecific and congeneric pairs. The evolutionary rate was quite homogeneous at both intraspecific and congeneric level, and the lowest congeneric rates were found in cryptic (morphologically undistinguishable) and in morphologically very similar species pairs. Moreover, congeneric nonsynonymous rates (d*N*) were up to two orders of magnitude higher than in intraspecies pairs. Overall, a clear-cut gap sets apart conspecific from congeneric pairs. These evolutionary peculiarities allowed easily identifying an extraordinary intraspecific variability in the model ascidian *Botryllus schlosseri*, where most pairs show a d*N* value between that observed at intraspecies and congeneric level, yet consistently lower than that of the *Ciona intestinalis* cryptic species pair. These data suggest ongoing speciation events producing genetically distinct *B. schlosseri* entities. Remarkably, these ongoing speciation events were undetectable by the *cox1* barcode fragment, demonstrating that, at low phylogenetic distances, the whole mitogenome has a higher resolving power than *cox1*. Our study shows that whole-mitogenome comparative analyses, performed on a suitable sample of congeneric and intraspecies pairs, may allow detecting not only cryptic species but also ongoing speciation events.

## Introduction

Ascidians, or sea squirts, are the largest and most diverse class of Tunicata, the chordate subphylum of marine filter-feeding organisms that for a long time is fascinating biologists for the extraordinary association of high morphoanatomical (adult) diversity and close evolutionary affinities to vertebrates. In addition to being a traditional target of developmental and embryological studies, the interest toward ascidians has recently grown up and expanded to new fields, also thanks to the advances in molecular biology and genomics. Thus, ascidians are emerging as model organisms for the study of processes as diverse as heart development, evolution of the immune system, conservation of gene regulatory networks in chordates, differentiation of specific cell lines and the respective molecular mechanisms, transcriptional control of embryonic development, comparative genomics, and so forth (Holland and Gibson-Brown 2003; Lemaire et al. 2008; Lemaire 2011; Tolkin and Christiaen 2012). As an example, the cosmopolitan and invasive colonial ascidian *Botryllus schlosseri* has become a model system for the study of natural transplantation reactions, apoptosis, stem cell-mediated regeneration, and asexual reproduction (Manni et al. 2007). Therefore, the recently sequenced nuclear genome of *B. schlosseri* (Voskoboynik et al. 2013) and its comparison with the genomes of other model tunicates, that is, the *Ciona intestinalis* and *C. savignyi* solitary ascidians and the larvacean *Oikopleura dioica* (Dehal et al. 2002; Small et al. 2007; Denoeud et al. 2010), promises new insights into the earlier-mentioned processes as well as into the deuterostome evolution and the origin of chordates.

In fact, most of the interest toward ascidians and tunicates can be brought back to their key phylogenetic position within Chordata, and the recently discovered monophyletic grouping of tunicates and vertebrates with the exclusion of cephalochordates. This finding, initially based on molecular evidences (Bourlat et al. 2006; Delsuc et al. 2006; Putnam et al. 2008) and then confirmed by some morphological data (Jeffery 2007), is now widely accepted and contributes to make tunicates in general, and ascidians in particular, invaluable chordate invertebrate model organisms. However, it should be noted that the ambiguities present in some nodes of the tunicate phylogenetic tree can reduce the reliability and the power of these organisms as models, because the lack of clear phylogenetic data could complicate the interpretation of comparative studies carried out both within tunicates and between tunicates and vertebrates.

It is noteworthy that debated phylogenetic issues in tunicates involve both high and low taxonomic ranks. At high taxonomic level, the most remarkable questions are the branching pattern between the three tunicate classes (Thaliacea, Larvacea, and Ascidiacea), the correlated issue of the Ascidiacea paraphyly (Wada 1998; Stach and Turbeville 2002; Zeng and Swalla 2005; Yokobori et al. 2006; Zeng et al. 2006; Swalla and Smith 2008; Tsagkogeorga et al. 2009; Govindarajan et al. 2011), and the Phlebobranchia paraphyly (Swalla et al. 2000; Turon and Lopez-Legentil 2004; Yokobori et al. 2005; Zeng and Swalla 2005; Tsagkogeorga et al. 2009; Stach et al. 2010; Rubinstein et al. 2013). At low taxonomic level, exemplifying cases are the relationships among the Aplousobranchia families (Turon and Lopez-Legentil 2004; Tsagkogeorga et al. 2009), the paraphyly of Pyuridae with respect to Styelidae (Perez-Portela et al. 2009; Rubinstein et al. 2013), the possible inclusion of Cionidae within Aplousobranchia rather than Phlebobranchia (Kott 1990; Stach and Turbeville 2002; Turon and Lopez-Legentil 2004), up to the existence of cryptic species in several ascidians (Tarjuelo et al. 2001, 2004; Perez-Portela and Turon 2008). Cryptic speciation has been reported even in the model organisms *C. intestinalis* (Suzuki et al. 2005; Caputi et al. 2007; Iannelli, Pesole, et al. 2007; Nydam and Harrison 2007, 2010; Zhan et al. 2010) and *B. schlosseri* (Bock et al. 2012).

The resolution of these issues through molecular phylogenetic studies is, however, complicated by the high nucleotide substitution rate found in all tunicates so far analyzed, both at level of nuclear and mitochondrial (mt) genes (Singh et al. 2009; Tsagkogeorga et al. 2010; Rubinstein et al. 2013 and references therein). Indeed, the fast evolutionary dynamics seems to be a pervasive, likely ancestral, tunicate feature, that affects even the overall organization and structural features of the nuclear and mitochondrial genomes (Yokobori et al. 2005; Gissi et al. 2008; Lemaire et al. 2008; Gissi et al. 2010; Rubinstein et al. 2013). The unresolved phylogenetic questions and the fast evolutionary dynamics of tunicates can be quite easily investigated in the small mt genome, which represents an attractive model system. In fact, the ascidian mitogenomes have already proved to be hypervariable in many genomic features, because they show very high sequence divergence, variable tRNA gene content, variable position of the longest noncoding region (NCR), and rampant gene order rearrangements even in congeneric and cryptic species (Iannelli, Pesole, et al. 2007; Gissi et al. 2008, 2010).

In the framework of a wider project on the phylogeny and mitogenomics of tunicates, we are sequencing the complete mitogenomes of several ascidians belonging to the three major groups of Aplousobranchia, Phlebobranchia, and Stolidobranchia. Here, we analyze 13 new mitogenomes of congeneric and conspecific samples to study the nucleotide substitution rate of ascidians at short phylogenetic distances. As novelty, the nucleotide substitution rate has been compared among different congeneric species as well as among different individuals of the same species, and also along the whole mitogenome, considering separately the different functional regions of this molecule (i.e., the different gene categories as well as the different codon positions and the NCRs). Thanks to the overall picture of the evolutionary rate inferred from this data set, we have been able to easily identify a surprisingly high intraspecific variability in the invasive and model species *B. schlosseri*. Indeed, the intraspecies and congeneric comparisons give strong indications of the existence in *B. schlosseri* of subtle ongoing speciation events, likely not yet corresponding to the emergence of fully differentiated species. Thus, here we demonstrate that our comprehensive comparative approach at low phylogenetic distance, consisting in the detailed and accurate comparison of the mitochondrial nucleotide substitution rate between several congeneric and intraspecies ascidian pairs, can help solving phylogenetic controversies at low taxonomic level, even in the absence of significant differences in the overall mitochondrial genome structure such as those found in the cryptic *C. intestinalis* species (Iannelli, Pesole, et al. 2007).

## Materials and Methods

### Mitochondrial Genome Sequencing

The 13 mitogenomes sequenced in this study are listed in table 1, together with the GenEMBL AC numbers. Except for *B. schlosseri*, the new mitogenomes will be described in detail in a distinct manuscript (Griggio F, Gissi C, in preparation).

**Table 1:** Species Classification and Accession Number (AC) of the Mitochondrial Genomes Analyzed in This Study

Classification	Species, Sample Name	AC Number	mtDNA (bp)
Stolidobranchia			
Styelidae, Botryllinae	*Botrylloides nigrum*^a^	HF548559	14,427
Styelidae, Botryllinae	*Botrylloides leachii*, BA_TR	HG931921^b^	14,408
Styelidae, Botryllinae	*Botrylloides leachii*, L2_VE	HF548553^b^	14,408
Styelidae, Botryllinae	*Botrylloides pizoni*, PE	HG931922^b^	14,323
Styelidae, Botryllinae	*Botrylloides pizoni*, VI	HF548554^b^	14,323
Styelidae, Botryllinae	*Botrylloides violaceus*	HF548552^b^	14,357
Styelidae, Botryllinae	*Botryllus schlosseri*, EA	HG931923^b^	14,934
Styelidae, Botryllinae	*Botryllus schlosseri*, sc6ab	HF548551	14,928
Styelidae, Botryllinae	*Botryllus schlosseri*, TR	HF548550^b^	14,932
Styelidae, Botryllinae	*Botryllus schlosseri*, VE	FM177702^b^	14,945
Styelidae	*Styela clava*	HG931920^b^	14,616
Styelidae	*Styela plicata*	AM292601	14,414
Pyuridae	*Halocynthia papillosa*	FM177863^b^	14,897
Pyuridae	*Halocynthia roretzi*	AB024528	14,771
Pyuridae	*Halocynthia spinosa*	HF548558	15,074
Phlebobranchia			
Cionidae	*Ciona intestinalis* sp.A, CAhm	AABS01001113	14,140^c^
Cionidae	*Ciona intestinalis* sp.A, ITna	AJ517314	14,790
Cionidae	*Ciona intestinalis* sp.B	AM292218	14,591
Cionidae	*Ciona savignyi*, CAsf	AACT01048180	14,732
Cionidae	*Ciona savignyi*, JPmb	AB079784	14,737
Ascidiidae	*Phallusia fumigata*	AM292602	15,535
Ascidiidae	*Phallusia mammillata*	AM292320	14,579
Aplousobranchia			
Polyclinidae	*Aplidium coeruleum*^d^	^b^	13,959^c^
Polyclinidae	*Aplidium conicum*	FN313538	15,183
Polyclinidae	*Aplidium tabarquensis*	HF548555^b^	8,117^c^
Clavelinidae	*Clavelina lepadiformis*, ITgl	AM292603	14,461
Clavelinidae	*Clavelina lepadiformis*, SEgu	FJ839918	14,484
Clavelinidae	*Clavelina phlegraea*	AM292604^b^	14,603

^a^Sequence originally reported as belonging to *Botrylloides affinis leachii* (Rubinstein et al. 2013).

^b^Sequences published in this study.

^c^Partial genomes.

^d^Genome assembled in eight contigs.

Ascidian samples were stored in 95% ethanol or RNA later until DNA extraction. Total DNA was extracted from the muscle of a single individual (for *Styela clava* and *Halocynthia papillosa*), from zooids previously separated from the tunic (for *Aplidium coeruleum* and *B. schlosseri* EA), or from a small piece of colony with the tunic (for all other colonial samples).

For *S. clava*, *A. coeruleum*, and *B. schlosseri* EA, total DNA was extracted using the DNeasy Plant Mini kit (QIAGEN) following the manufacturer’s protocol. Genomic DNA libraries were prepared with the blunt-end repair, A-tailing, adapter ligation, fill-in, and indexing steps (Meyer and Kircher 2010). Libraries were pooled in equimolar quantities for a total combined quantity of 2 µg in a final volume of 100 µl. Illumina single reads sequencing was completed by the GATC–Biotech company (Konstanz, Germany) on one lane of HiSeq 2000. The mitogenomes of these three samples were assembled from these Illumina reads according to the strategy described in Botero-Castro et al. (2013).

For the remaining ten specimens, the total DNA was extracted using the Puregene Tissue kit (Gentra Systems) for *H. papillosa* and a modified CTAB method (Hirose M and Hirose E 2009) for colonial specimens processed with the tunic. The mitogenomes were then produced according to a standard strategy (Gissi et al. 2004; Iannelli, Griggio, et al. 2007; Gissi et al. 2010), consisting in the amplification of the whole mitogenome in a few long overlapping fragments followed by Sanger sequencing. All amplicons were produced using high fidelity polymerases or polymerase mixs, to reduce the risk to introduce nucleotide substitutions during the amplification steps. Polymerase chain reaction (PCR) reactions were initially carried out using previously published (Iannelli, Griggio et al. 2007; Gissi et al. 2010) and new heterologous primers, manually designed on the most conserved mt genes (*cob*, *cox1*, *cox2*, *cox3*, *rrnL*, and *rrnS*) based on the alignment of several deuterostome representative species (including all available ascidian sequences). Given the absence of information on the mt gene order in the analyzed species, several combinations of these heterologous primers were tried in the initial PCR reactions, and only those reactions that gave a bright single band during electrophoretic analysis were further processed. If necessary, species-specific primers were designed on the sequences of the initially obtained amplicons, and used to amplify the remaining portion(s) of the mt genome. Depending on size and yield, amplicons were completely sequenced by primer walking, or used as template in nested/seminested PCRs to obtain short sequenceable fragments. Amplicons with long homopolymeric tracts, producing low-quality sequences, were cloned using the CloneJET PCR (Fermentas) or the TOPO-TA (Invitrogen) cloning kit, and their final sequence was set to the consensus of three clones. The cloning strategy, related to the presence of homopolymeric tracts, was necessary in all analyzed species except for *Botrylloides*. Sanger sequencing was performed by the Eurofins MWG operon company (Ebersberg, Germany).

For each species, the list of the amplicons covering the entire mitogenome and the sequences of the used heterologous and species-specific primers are reported in supplementary table S1, Supplementary Material online. In general, amplicon size ranged from 4 to 9 kb but amplicons ≤2.5 kb were also produced to confirm/obtain the overlap between the longest amplicons. As an exception, the mitogenome of the *Botrylloides pizoni* PE specimens was amplified in a total of 11 fragments, with a maximum size of 2.7 kb. This strategy was necessary because of the poor conservation state of this sample and the consequent high fragmentation of the extracted DNA. As for *A. tabarquensis*, we were able to sequence only about half of its mitogenome, despite the numerous attempts to amplify the remaining mt region using species-specific primers.

### Gene Annotations

Mitochondrial genes were annotated by similarity to orthologous metazoan genes, taking advantage of the BlastN/BlastP service of the MitoZoa database (D'Onorio de Meo et al. 2012). The start codon of protein-coding genes (PCGs) was defined as the first ATG or nonstandard initiation codon (Wolstenholme 1992), not in overlap with the upstream gene and maximizing the similarity to orthologous ascidian proteins. According to the existence of a tRNA punctuation model in the ascidian mt transcript maturation (Gissi and Pesole 2003), incomplete T or TA stop codons were hypothesized only if immediately adjacent to a downstream tRNA gene, and then assumed to be completed by posttranscriptional polyadenylation.

Transfer RNA genes were identified by their potential cloverleaf secondary structure using the programs tRNAscan-SE (Lowe and Eddy 1997) and ARWEN (Laslett and Canback 2008). tRNAs with unusual structure, such as those lacking an arm, were searched also using specific patterns designed with the PatSearch program (Pesole et al. 2000). All the earlier-predicted tRNA sequences were checked through multialignment to orthologous tRNAs of other ascidians and deuterostome representatives. Therefore, the final tRNA boundaries were defined based on sequence similarity and on the presence of a conserved cloverleaf secondary structure.

tRNA genes were named according to the common nomenclature of mt tRNA genes, and to the MitoZoa database (D'Onorio de Meo et al. 2012). Thus, only the isoacceptor tRNA genes for Gly, Ser, and Leu were named based on the recognized codons. Moreover, the two isoacceptor tRNA genes for Met were named based on the anticodon sequence, due to the absence of functional data on possible differences in the recognized codons. The boundaries of the two rRNA genes were inferred as abutted to the flanking genes.

### Comparative Analyses of the Whole Mitogenomes

Comparative analyses at intraspecies and congeneric levels were carried out on the 28 mitogenomes listed in table 1. The exact source and sampling date of the specimens analyzed in intraspecies comparisons are reported in table 2. The mt scaffolds of *C. intestinalis* sp.A and *C. savignyi* listed in table 2 derive from the whole-genome shotgun (WGS) projects of these species and were already described in Iannelli, Pesole, et al. (2007). The mt sequence of *Botrylloides nigrum* (table 2) was originally published as belonging to *Botrylloides* *affinis leachii* (Rubinstein et al. 2013) but a reanalysis of the morphology of the voucher specimen (Shenkar N, personal communication), together with molecular analyses on additional samples (our data), has allowed its reassignment to *Botrylloides* *nigrum*, a species that can be easily morphologically confused with *Botrylloides leachii* (Brunetti 2009).

**Table 2:** Data on the Ascidian Mitochondrial Genomes Analyzed in This Study in Intraspecies Comparisons

Species	Name	Source	Method (Notes)	AC Number	mtDNA (bp)	Total NCR (bp)
*Botryllus schlosseri*	VE	Venice Lagoon, Northern Adriatic Sea, Italy (July 2005)	Long-accurate PCR + Sanger	FM177702^a^	14,945	866
	sc6ab	Santa Cruz Harbour, Pacific Ocean, California (laboratory-maintained)	Scaffold of WGS + PCR and Sanger	HF548551	14,928	838
	TR	Taranto Gulf, Northern Ionian Sea, Italy (September 2011)	Long-accurate PCR + Sanger	HF548550^a^	14,932	838
	EA	Els Alfacs bay, Ebre Delta, Western Mediterranean coast, Spain (January 2006)	Illumina sequencing	HG931923^a^	14,934	838
*Botrylloides leachii*	L2_VE	Venice Lagoon, Northern Adriatic Sea, Italy (December 2010)	Long-accurate PCR + Sanger	HF548553^a^	14,408	246
	BA_TR	Taranto Gulf, Northern Ionian Sea, Italy (September 2011)	Long-accurate PCR + Sanger	HG931921^a^	14,408	248
*Botrylloides pizoni*^b^	VI	Taranto Gulf, Northern Ionian Sea, Italy (September 2011)	Long-accurate PCR + Sanger	HF548554^a^	14,323	155
	PE	Harbour Island, San Diego Bay, Pacific Ocean, California (June 2012)	Long-accurate PCR + Sanger	HG931922^a^	14,323	155
*Ciona intestinalis sp.A*	ITna	Gulf of Naples, Italy	Long-accurate PCR + Sanger	AJ517314	14,790	447
	CAhm	Half Moon Bay, California	Scaffold of WGS (unsequenced *trnW* and part of *rrnS* and *nad6*)	AABS01001113	14,140	451
*Ciona savignyi*	JPmb	Mutsu Bay, Japan	Long-accurate PCR + Sanger	AB079784	14,737	427
	CAsf	San Francisco Bay, California	Scaffold of WGS	AACT01048180	14,732	428
*Clavelina lepadiformis*	ITgl	IT: Gulf of La Spezia, Italy	Long-accurate PCR + Sanger	AM292603	14,461	315
	SEgu	Gullmarsfjord, Sweden	Long-accurate PCR + Sanger	FJ839918	14,482	282

^a^Sequences published in this study.

^b^*Botrylloides pizoni* sensu Brunetti and Mastrototaro (2012).

The 13 PCGs and the two rRNAs were initially aligned with MATTF v6 (Katoh and Toh 2008) and then the alignments were manually optimized. Protein alignments were back-translated to the nucleotide level using TranslatorX (http://translatorx.co.uk/, last accessed March 11, 2014). tRNA genes were manually aligned based on the previously defined secondary structures. Finally, for each gene category (PCG, tRNA, and rRNA), a concatenated alignment was produced with Geneious (http://www.geneious.com/, last accessed March 11, 2014) and used in sequence divergence calculations.

The nonsynonymous (d*N*) and synonymous (d*S*) pairwise substitutions rates were calculated with the codeml program of the PAML v4.4b package (Yang 1997), using an advanced model of codon substitution accounting for differences in the transition–transversion rates and for the codon usage bias (Goldman and Yang 1994). The codeml program was run with the options CodonFreq = 2, icode = 9 (ascidian mt) and runmode = −2 (pairwise comparison). Pairwise uncorrected distances were calculated using PAUP*, separately for the first plus second codon positions (P12), and the third codon position (P3) (Swofford 2003). Saturation analyses were then carried out plotting the PAUP* uncorrected distances versus the inferred number of substitutions calculated with PAML.

Congeneric comparisons were carried out only at level of synonymous and nonsynonymous sites, because these sites constitute most of the mt genome and because of the availability of appropriate evolutionary models allowing accurate d*N* and d*S* calculations (Goldman and Yang 1994). Moreover, for some congeneric pairs no reliable alignments can be obtained at level of NCR and of some rRNA regions.

### Analyses of Cox1 Sequences of *B. schlosseri*

To further investigate the *B. schlosseri* intraspecies variability, we analyzed all 138 partial *cox1* sequences of *B. schlosseri* available in GenEMBL at May 2013. A compilation of *cox1* sequences belonging to closely related species was also considered (GenEMBL, May 2013), to select the most reliable *B. schlosseri* outgroup through preliminary phylogenetic reconstructions. Thus, the analyzed outgroup sequences were as follows: *Botrylloides violaceus* (18 sequences), *Botrylloides leachii* (3 seq), *Botrylloides pizoni* (2 seq), *Botrylloides fuscus* (1 seq), *Botrylloides nigrum* (1 seq), *B. tyreus* (a synonymous of *Botrylloides tyreum*, see the WORMS database at http://www.marinespecies.org/ (last accessed March 11, 2014); 1 sequence), *Symplegma rubra* (1 seq), *S. plicata* (1 seq), and *S. clava* (1 seq). The full list of the analyzed *cox1* sequences is reported in supplementary table S2, Supplementary Material online, together with specimen source and sequence identity.

The *cox1* sequences were aligned at amino acid level with MATTF v6 (Katoh and Toh 2008), and then back-translated into a nucleotide alignment. Sequences too short compared with the others, or identical within a given species (i.e., corresponding to the same haplotype), were eliminated before the phylogenetic reconstructions. Preliminary maximum likelihood (ML) trees were used to select the best outgroup to *B. schlosseri*, that is, the species showing the shortest genetic distance to the ingroup. The final *cox1* alignment is 522-bp long and corresponds exactly to the *cox1* “DNA barcode” fragment analyzed by Bock et al. (2012). This alignment includes 71 *B. schlosseri* sequences and the three best outgroup sequences belonging to *Botrylloides* *leachii* and *Botrylloides* *nigrum*.

The phylogenetic reconstructions were carried out with PhyML v3.0 (Guindon et al. 2010). The evolutionary models best fitting to the analyzed alignments were selected among 88 different models with jModelTest v2.1.3, using an ML optimized tree for likelihood calculations and according to the Akaike Information criterion (Guindon and Gascuel 2003; Darriba et al. 2012). The model best fitting to our final *cox1* alignment was TIM3 + G (with G indicating a gamma distribution for rate heterogeneity across sites).

To investigate the substitution saturation of *cox1*, for all sequence pairs of the data set, we plotted the PAUP* uncorrected distances versus the inferred number of substitutions calculated as PhyML patristic distances (i.e., the sum of the lengths of all branches linking two sequences on the PhyML tree). The PATRISTIC program (Fourment and Gibbs 2006) was used to extract the patristic distances from the PhyML tree.

## Results and Discussion

### Mitogenome Data Set

Using two different approaches, that is, a conventional long PCR strategy followed by Sanger sequencing and an advanced next-generation sequencing of a total DNA extract, we have sequenced a total of 13 mitogenomes: 11 complete genomes, belonging to 7 different species, and two partial genomes belonging to two *Aplidium* species (table 1). Together with other available congeneric and conspecific mitogenomes, the new sequences constitute a suitable data set for investigating the intraspecies and congeneric variability of ascidians. Indeed, this data set allows conspecific and congeneric analyses in six different species and seven different genera, respectively (table 1).

For intraspecies comparisons, only specimens sampled in distant localities and/or in different years have been taken into account (table 2), to be reasonably confident that they belong to different populations. As for congeneric comparisons, our data set includes some noteworthy species, such as:

- representatives of both genera, *Botryllus* and *Botrylloides*, forming the subfamily Botryllinae;
- the two cryptic species of *C. intestinalis*, named sp.A and sp.B (Suzuki et al. 2005; Caputi et al. 2007; Iannelli, Pesole, et al. 2007; Nydam and Harrison 2007, 2010; Zhan et al. 2010);
- *Botrylloides* *leachii* and *Botrylloides* *nigrum*, that is two *Botrylloides* species that are morphologically very similar and are discriminated mainly based on the mode of larva incubation (Brunetti 2009).

Finally, the analyzed intraspecies and congeneric comparisons concern species belonging to all three major ascidian groups of Aplousobranchia, Phlebobranchia, and Stolidobranchia, and thus they cover a wide phylogenetic range (tables 1 and 2).

### Intraspecies Divergence

**Fig. 1.—:**
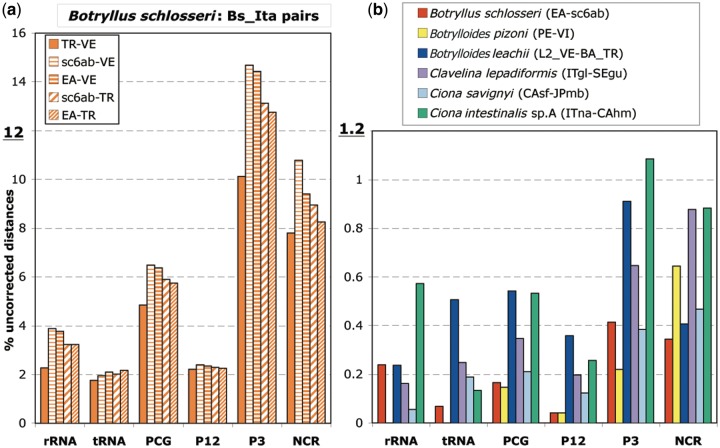
Uncorrected distances (in %) of 11 intraspecies comparisons, calculated on the various functional regions of the mitochondrial genome. Note the different scale used in the two panels. (*a*) The four *Botryllus schlosseri* intraspecies pairs involving at least one of the two VE and TR Italian specimens (Bs_Ita pairs). (*b*) The EA-sc6ab *B. schlosseri* pair and all other intraspecies comparisons. P12: first and second codon positions; P3: third codon position. The analyzed mitogenomes are listed in table 2, together with specimen abbreviation and source data.

For the ascidian species with at least two available mitogenomes, the intraspecies uncorrected distances were calculated separately for the different functional mt regions, namely the various gene categories (rRNAs, tRNAs, and PCGs), the first plus second codon positions (P12), the third codon position (P3), and the concatenation of the few NCR (fig. 1). Surprisingly, the pairwise uncorrected distances within *B. schlosseri* (four samples listed in table 2) are extraordinarily higher in the five pairs involving at least one of the two (TR and VE) Italian specimens (hereafter named Bs_Ita pairs) than in the comparison between the Spanish (EA) and the Californian (sc6ab) *B. schlosseri* samples. Therefore, figure 1 has been split in two distinct graphs, each using a different scale: figure 1*a* shows only the uncorrected distances of the *B. schlosseri* Bs_Ita comparisons, while figure 1*b* shows the uncorrected distances of the EA-sc6ab *B. schlosseri* pair and of all remaining intraspecies pairs. Remarkably, the uncorrected distances of the Bs_Ita pairs are up to one order of magnitude higher than those of all other intraspecies comparisons and of the *B. schlosseri* EA-sc6ab pair (from 4 to 59 times higher, depending on the analyzed mt region). Taking into account just the PCGs, that form about 75% of the whole mitogenome, the *B. schlosseri* Bs_Ita pairs evolve on average 35 times faster than the EA-sc6ab pair and 11–40 times faster than other intraspecies pairs (cf. fig. 1*a* with fig. 1*b*). Thus, based on the intraspecies sequence divergence, we can recognize within *B. schlosseri* three distinct clusters (EA + sc6ab, VE, and TR) that could correspond to cryptic species or to a still ongoing speciation event. It should be noted that the existence of cryptic species in *B. schlosseri* has been already proposed based on the analyses of nuclear and mitochondrial markers (Bock et al. 2012). However, as discussed later, our specimens belong to the same *B. schlosseri* cryptic species sensu Bock et al. (2012) (see the paragraph “*cox1* analyses”). The low sequence divergence between samples coming from very distant localities (i.e., EA from Spain and sc6ab from California) can be explained by the widely ascertained invasive nature of this species (Lambert CC and Lambert G 1998; Lambert 2001; Carlton 2005; Carver et al. 2006; Lejeusne et al. 2011).

As for the other analyzed ascidians, the lowest intraspecies difference is observed within *Botrylloides pizoni* sensu Brunetti and Mastrototaro (2012) that shows even no nucleotide substitutions in the tRNA and rRNA genes (absence of yellow bars in rRNA and tRNA data of fig. 1*b*). As expected, in each species the fastest evolving mt regions are the P3 or NCR, which evolve from 1.7 up to 15.6 times faster than the P12, rRNAs, and tRNAs of the same species (fig. 1*a* and *b*).

### Congeneric Divergence in the PCGs

To clarify the relevance of the high intraspecies sequence variability found in *B. schlosseri*, we have compared the d*S* and d*N* nucleotide substitution rates in several intraspecies and congeneric ascidian pairs (PAML calculations, see Materials and Methods). Assuming that congeneric species are the most closely related organisms above the species level, we should observe a considerable difference and a clear-cut gap between the d*N* and d*S* values calculated at intraspecies and at congeneric level. It should be noted that our data set includes three different species of *Botrylloides*, a genus closely related to *Botryllus*, so it can be considered adequate to analyze the evolutionary rate within Botryllinae.

The d*S* average value of the *B. schlosseri* Bs_Ita pairs is one order of magnitude higher than those of other intraspecies comparisons (0.30 ± 0.06 substitutions/site against 0.06 for EA-sc6ab, and 0.013 ± 0.008 for other species). Moreover, no evidence of d*S* saturation is observed at intraspecies level (d*S* < 1 substitution/site and saturation plot results, data not shown), while the d*S* is fully saturated in all congeneric comparisons and even in the cryptic *Ciona* species pairs (d*S* values > 1 substitution/site and saturation plot results, data not shown; d*S* range: 3.2–3.4 in the cryptic and *Botrylloides leachii*–*Botrylloides nigrum* species pairs, and 5.3–170.9 in other congeneric pairs). Overall, the d*S* data confirm the surprisingly high substitution rates of the *B. schlosseri* Bs_Ita pairs relative to all other intraspecies pairs; however, the dS saturation at congeneric level does not allow a reliable quantification of the substitution rate differences between intraspecies and congeneric pairs.

**Fig. 2.—:**
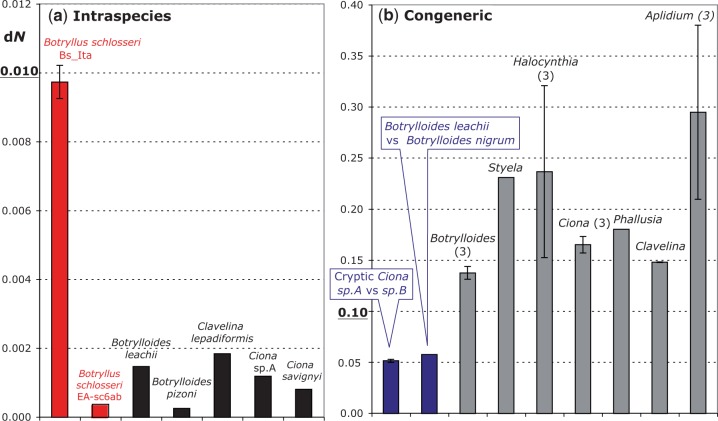
Mean and standard deviation of the nonsynonymous substitution rates (d*N*) inferred for: (*a*) intraspecies and (*b*) congeneric ascidian pairs. Red: *Botryllus schlosseri* comparisons; blue: comparisons between cryptic or morphologically very similar species pairs. The number of pairs examined for each genus is reported in brackets, if >1. The analyzed mitogenomes are listed in table 1. d*N* rates were calculated with PAML v4.4b package (Yang 1997) according to Goldman’s codon substitution model (Goldman and Yang 1994).

**Fig. 3.—:**
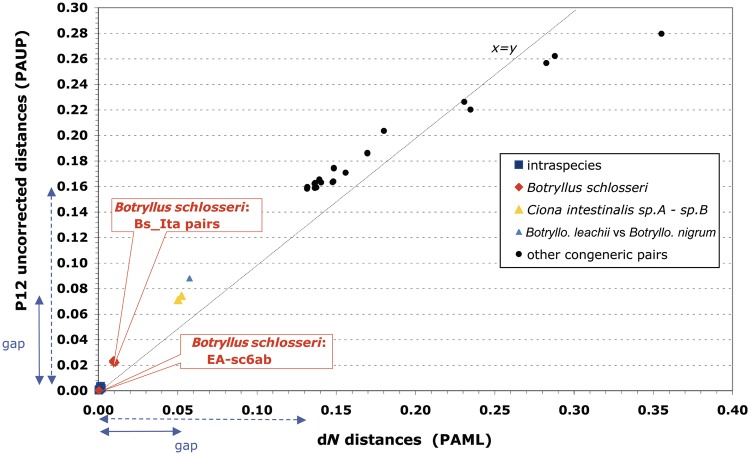
Saturation plot of the first plus second codon positions (P12) of the 13 mitochondrial protein-genes, drawn for all congeneric and intraspecies ascidian pairs. Continuous violet arrowhead lines: gap between intraspecies and all congeneric distances. Dotted violet arrowhead lines: gap between intraspecies and congeneric distances excluding the cryptic *Ciona intestinalis* and the *Botrylloides leachii–Botrylloides nigrum* species pairs. The “*x* = *y*” line represents the situation where the number of inferred substitutions is equal to the number of observed differences.

The d*N* rates of the analyzed intraspecies and congeneric comparisons are shown in two distinct panels of figure 2, each using a different scale. In each panel, almost all values have the same order of magnitude and are well comparable: the only exception is again provided by the surprisingly high d*N* values of the *B. schlosseri* Bs_Ita pairs (red bar in fig. 2*a*), which are 5–39 times higher than that found in other intraspecies comparisons but still 5–30 times lower than that of congeneric pairs (fig. 2*b*). Thus, the d*N* values of these exceptional *B. schlosseri* Bs_Ita pairs are exactly in between those commonly observed in intraspecies and congeneric pairs. This is also evident in the d*N* saturation plot of figure 3, which not only demonstrates the absence of nonsynonymous saturation at both intraspecies and congeneric level but also clearly shows that the d*N* values of the Bs_Ita pairs are considerably above all other intraspecies values and below the lowest interspecies d*N* values observed in the cryptic *C. intestinalis* sp.A–sp.B species and in the two morphologically very close species *Botrylloides leachii* and *Botrylloides. nigrum* (see triangles in fig. 3 and blue bars in fig. 2). These data suggest that the high intraspecies sequence divergence observed in our *B. schlosseri* specimens is compatible with an ongoing speciation process or with the presence of genetically separated cryptic species.

The conspecific and congeneric comparisons here analyzed highlight the existence of a substantial gap between intraspecies and congeneric d*N* rates, that becomes even wider when the congeneric data set is deprived of the cryptic and morphologically very similar species pairs (fig. 3). In particular, excluding the *B. schlosseri* Bs_Ita pairs, the delta between the highest intraspecies and the lowest interspecies d*N* values ranges from 0.05 for the whole congeneric data set, to 0.13 for a reduced data set deprived of the pairs *C. intestinalis* sp.A–sp.B and *Botrylloides leachii*–*Botrylloides nigrum* (violet arrowed lines in fig. 3). It can be also noted that, without the *B. schlosseri* Bs_Ita pairs, the d*N* rate is relatively constant at both intraspecies and interspecies level, because it varies only 7 times at intraspecies level, and 3–7 times at congeneric level depending on the exclusion/inclusion in the data set of the cryptic *Ciona* and *Botrylloides leachii*–*Botrylloides nigrum* pairs (fig. 2). As our data set includes species of all three major ascidian lineages, selected without any peculiar bias and only based on mitogenome availability (tables 1 and 2), we can assume that the intraspecies and congeneric d*N* ranges here estimated are representative of the ascidian variability. This observation further supports the exceptional character of the Bs_Ita pairs compared with both intraspecies and congeneric comparisons, and then the existence of ongoing/cryptic speciation events separating VE, TR, and the EA+sc6ab *B. schlosseri* specimens.

### *cox1* Analyses

*Botryllus schlosseri* has been the target of many population genetic studies based on the standard “DNA barcode,” that is a fragment of the mt *cox1* gene, about 650-bp long, hereafter referred as “*cox1* barcode-fragment” (Folmer et al. 1994; Lopez-Legentil et al. 2006; Lejeusne et al. 2011; Bock et al. 2012). Here, we have checked whether the intraspecies variability observed in our *B. schlosseri* samples through whole-mitogenome comparisons is detectable also by the analysis of the *cox1* barcode-fragment.

**Fig. 4.—:**
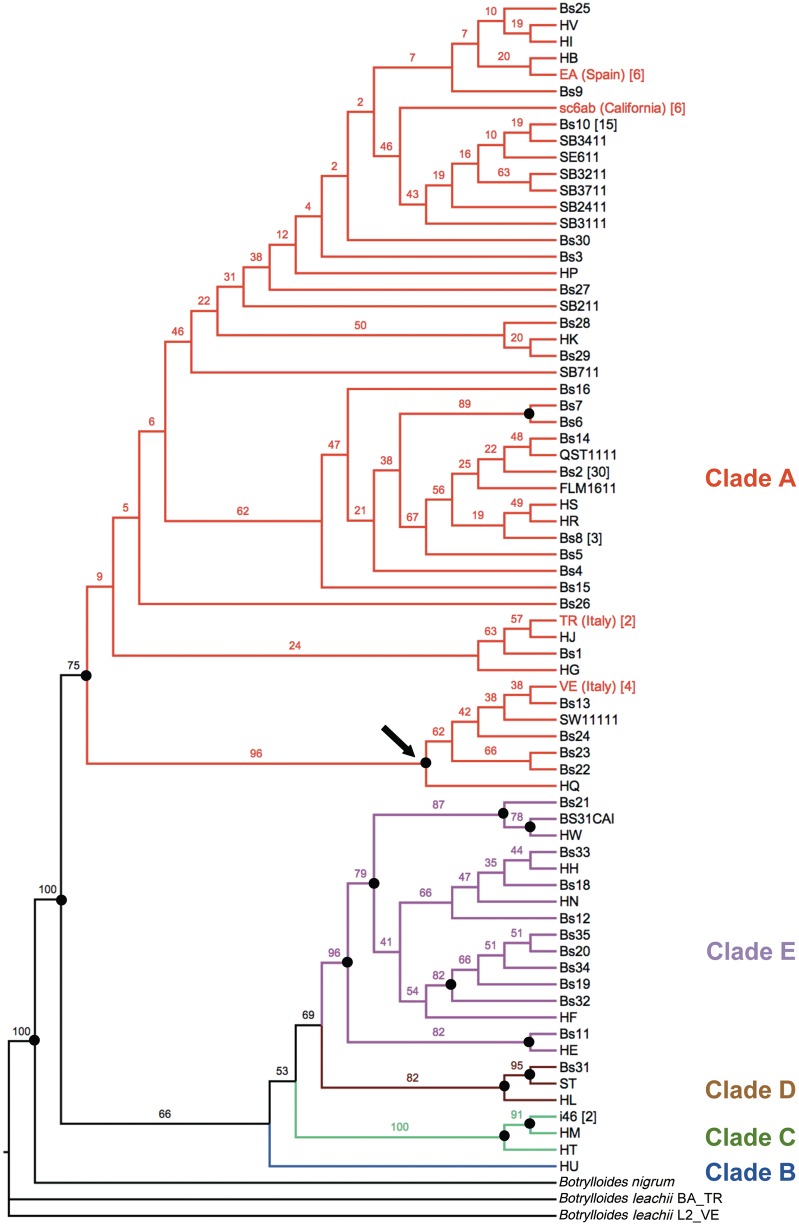
ML phylogenetic tree of *Botryllus schlosseri* calculated on the *cox1* barcode-fragment (PhyML: TIM3 + G model). Numbers at nodes indicate bootstrap support on 100 replicates; dots point to reliable nodes with bootstrap >70. The distinct clades, previously identified by Bock et al. (2012), are reported in different colors. Arrow points to the only highly supported large subclade within clade A. The number of GenEMBL *cox1* entries corresponding to a given haplotype is reported in square brackets, according to supplementary table S2, Supplementary Material online.

Our four *B. schlosseri* specimens have *cox1* sequences equal to haplotypes already sampled in other world localities (supplementary table S2, Supplementary Material online; see also square brackets in figure 4, reporting the number of *cox1* GenEMBL entries showing a given haplotype). The ML phylogenetic tree of all available *cox1* sequences of *B. schlosseri* (fig. 4) identifies only five well-supported groups, perfectly corresponding to the monophyletic A to E clades previously recognized by Bock as distinct and, probably, reproductively isolated cryptic species (Bock et al. 2012). This data set does not show substitution saturation in all (supplementary fig. S1, Supplementary Material online) as well as in the third codon positions (data not shown), but the slope of the regression line is very different for the intraclade and the interclade comparisons. Moreover, there is a wide gap between intraclade and interclade distances (supplementary fig. S1 and phylogram of supplementary fig. S2, Supplementary Material online), a feature that reminds the well-known “DNA barcode gap” (Goldstein and DeSalle 2011). Thus, the deep genetic separation observed between these clades, coupled with inconsistencies in the morphological description of *B. schlosseri* (Boyd et al. 1990), could also indicate that each of these clades corresponds to closely related but distinct species, all incorrectly assigned to *B. schlosseri* due to the intrinsic difficulties in the morphological identification/description of this species*.*

In the *cox1* phylogenetic tree of figure 4, all our *B. schlosseri* specimens fall within clade A, previously described as the most common and widespread *B. schlosseri* cryptic species (Bock et al. 2012). Interestingly, the *cox1* tree leaves unresolved almost all relationships within clade A, except for the identification of a highly supported subclade (96% bootstrap) including our VE specimen and few other haplotypes (see arrow in fig. 4). Therefore, the *cox1* barcode-fragment does not recognize the intraspecies differences that were clearly identified by the whole-mitogenome comparisons. We can hypothesize that the *cox1* barcode-fragment is unable to identify subtle, yet biologically significant, intraspecies differences due to its short length and the consequent low phylogenetic signal. In contrast, our comprehensive nucleotide substitution study carried out on the whole mitogenome captures this information quite easily, provided that sufficient intraspecies and congeneric pairs are available for comparative analyses.

To our knowledge, no other studies have compared the resolving power of the *cox1* barcode-fragment to that of the whole mitogenome. However, it is known that the *cox1* barcode is of limited usefulness not only in animals with slow-evolving mitogenomes, such as Cnidara and Porifera (Bucklin et al. 2011), but also in the fast-evolving mitogenomes of Amphibia, where the absence of a sharp distinction between intra- and interspecific divergence values complicates the clear establishment of a threshold for species discrimination (Vences et al. 2005).

### *Botryllus schlosseri* Mitogenome Structure

**Fig. 5.—:**
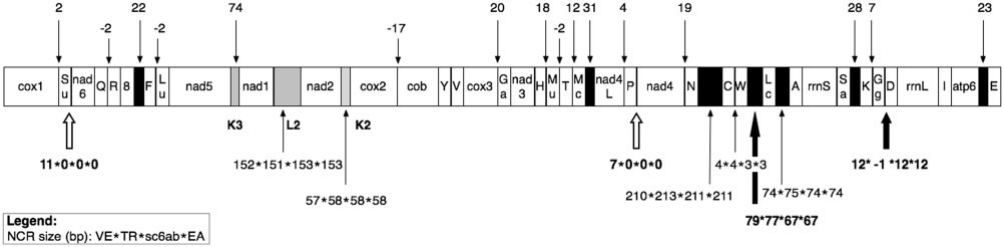
Mitochondrial genome organization of the four *Botryllus schlosseri* specimens, with size of the NCR. Black boxes: NCRs > 20 bp; gray boxes: NCRs > 20 bp containing a tRNA-like structure. Negative numbers indicate gene overlaps. NCRs with equal/different length in the four specimens are shown above and below the gene order diagram, respectively. Large black arrows: large NCR differences due to real indels. White arrows: large NCR differences due to the inclusion of a short sequence in a NCR or in a gene region, depending on the specimen. tRNAs are named by the transported amino acid (see also Materials and Methods). 8, atp8; Ga, tRNA-Gly(AGR); Gg, tRNA-Gly(GGN); Lu, tRNA-Leu(UUR); Lc, tRNA-Leu(CUN); Mc, tRNA-Met(CAU); Mu, tRNA-Met(UAU); Sa, tRNA-Ser(AGY); Su, tRNA-Ser(UCN).

To complete the *B. schlosseri* intraspecies analyses, we have carefully compared the mt genome structure of our four *B. schlosseri* specimens. All these mitogenomes contain the canonical tunicate gene complement (including the tunicate-specific *trnM(UAU)* and *trnG(GGN)* genes) and have identical gene order, with all genes encoded by the same strand (fig. 5). Only small differences have been observed in genome size (delta range: 4–17 bp) and total NCR length (delta: 29 bp) (table 2).

As shown in figure 5, differences in the length of NCRs/overlaps can be found at nine gene adjacencies but are sizeable only in four cases (fig. 5: bold number below the gene order diagram). Remarkably, these sizeable NCR differences are due to real indels only in two positions (indels of 11 and 13 bp at the adjacencies *trnW-trnL(CUN)* and *trnG(GGN)-trnD*, respectively; large black arrows in fig. 5). On the contrary, at the two other adjacencies (large white arrows in fig. 5) the NCR differences are due to the presence of a short sequence acting as NCR or as coding region depending on the specimen (11- and 7-bp long in *trnS(UCN)-nad6* and *trnP-nad4*, respectively). In particular, these last two cases are caused by the different size of a single large homopolymer (≥9 T) located just at the beginning of a protein gene (*nad6* and *nad4*, respectively) and by the consequent shift in the start codon of the protein-gene itself.

The NCR size variability found within *B. schlosseri* follows a pattern similar to that observed in the other analyzed intraspecies comparisons. Indeed, as shown in supplementary figure S3, Supplementary Material online, only few NCR size differences, always of small extent, have been found in some intraspecies comparisons (no intraspecies NCR differences have been found in *C. savignyi* and *Botrylloides pizoni*). Moreover, almost all these NCR differences are due to changes in the length of omopolymeric stretches located within or close to the same NCRs. For example, within *Clavelina lepadiformis* all NCR size differences are due to the variable length of one/more homopolymers (≥9 T) that cause the shift of the start/stop codon of the adjacent protein-gene (supplementary fig. S3, Supplementary Material online). Similarly, in *C. intestinalis* sp.A, NCR size differences are due to changes in the length of T or A homopolymers ≥6 nucleotides or in the number of dinucleotide repeats.

In conclusion, the structural features are (almost) identical between the four analyzed *B. schlosseri* mitogenomes, showing a situation not comparable with that previously observed in the *C. intestinalis* cryptic species, where significant changes in gene order, NCR and base composition have been found (Iannelli, Pesole, et al. 2007).

**Fig. 6.—:**
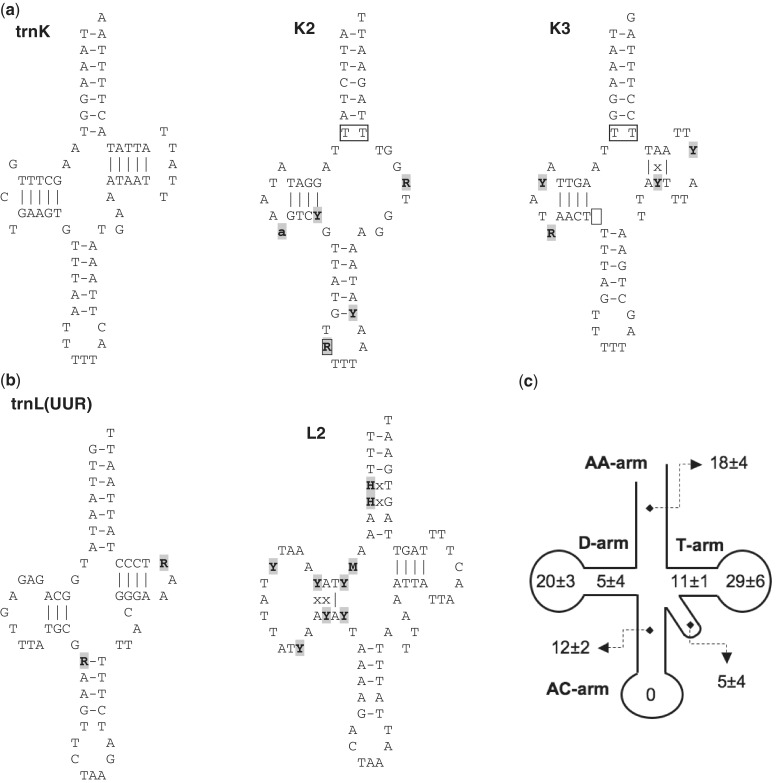
Secondary structures of *trnK*, *trnL(UUR)*, and three tRNA-like structures (K2, K3, and L2), together with the overall tRNA nucleotide substitution pattern within *Botryllus schlosseri*. (*a*) Secondary structure of *trnK* and the tRNA-like structures K2 and K3. (*b*) Secondary structure of *trnL(UUR)* and the tRNA-like structure L2. (*c*) Distribution of nucleotide substitutions in the different structural elements of the *B. schlosseri* tRNAs. Numbers indicate average and standard deviation of the pairwise nucleotide differences (in percentage), over all 24 tRNAs and in five *B. schlosseri* pairwise comparisons (the EA-sc6ab pair was excluded due to the almost identity between the tRNA sequences). Gray background: sites with nucleotide substitutions (upper case) or indels (lower case) in at least one of the six intraspecies *B. schlosseri* pairs. Borders: sites with uncommon features for functional tRNAs. “x”: nucleotide mispairing in at least one of the four specimens, due to noncompensatory substitutions.

As a peculiarity, all *B. schlosseri* mt genomes encode for three tRNA-like structures, named K2, K3, and L2 because they show, at the position corresponding to the possible anticodon, the sequence typical of the *trnK* or *trnL(UUR)* genes (figs. 5 and 6*a* and *b*). Although quite conserved at intraspecies level, these tRNA-like structures show several features quite uncommon in functional mt tRNAs (Kumazawa and Nishida 1993), which suggest that none of them is a functional tRNA. As shown in figure 6, these unusual features include the following:

- the lack of the T-arm (in K2),
- the absence of the nucleotide spacer between the D- and the anticodon (AC)-arm (in K3),
- the presence of a purine instead of a pyrimidine at the semi-invariant position just 5′ of the anticodon (in K2),
- the presence of a base mispairing at the basis of the amino acid acceptor (AA) stem (in K2 and K3),
- the presence of too many mispairing in the AA stem (in L2).

To obtain further hints on their possible functional role, we have also compared the overall evolutionary pattern of these three tRNA-like structures to that of the 24 canonical tRNAs of *B. schlosseri* (excluding the pair EA-sc6ab, because these sequences are almost identical at tRNA level). Although the level of nucleotide conservation is quite variable among the different tRNAs (% differences: 0–6.9% nucleotide differences), the identified tRNA-like structures evolve faster than most canonical tRNAs (1.6–10.4% nucleotide differences). As for the distribution of the nucleotide differences among the various stem/loop elements of a tRNA, in the canonical tRNAs most substitutions occur in the T-arm (40 ± 7%), mainly in the relative loop, followed, in almost equal measure, by the loop of the D-arm and by the AA-arm (18–20%) (fig. 6*c*). Remarkably, this trend is not observed in L2 and K2 that show an excess of substitutions in the D-arm (up to 67%) and AC-arm (up to 50%), respectively. Finally, in the canonical tRNAs, 90% of the total stem differences are compensatory substitutions, while in K3 and L2 there is no prevalence of compensatory substitutions. In conclusion, the overall evolutionary pattern of L2, K2, and K3 (fastest substitution rate, unusual distribution of the nucleotide substitution among the different stem/loop regions, and few compensatory substitution) does not support their role as tRNAs. However, the observed intraspecies conservation suggests an unknown function. Finally, the absence of significant similarity to other mt genes/regions prevents us from putting forward any hypothesis on their origin.

## Conclusions

In this study, we have performed congeneric and conspecific comparative analyses on a total of 28 ascidian mitogenomes, including 13 new mitogenomes, with the aim of studying the nucleotide substitution rate of ascidians at short phylogenetic distances. For the sake of accuracy, the substitution rate analyses have been carried out at whole-genome level considering separately the different functional regions of this molecule (i.e., the different gene categories as well the different codon positions and the NCRs). Although our taxon sampling is far from being comprehensive, both our intraspecies and congeneric data sets include species belonging to the main ascidian groups of Aplouso-, Phlebo-, and Stolido-branchia. Moreover, the comparisons between cryptic (*C. intestinalis* sp.A–sp.B) and morphologically very similar (*Botrylloides leachii*–*Botrylloides nigrum*) species add further validity to our data set, because they permit a more straightforward interpretation of the identified substitution rate differences. Thus, despite its small size, our data set can be considered quite solid for the aim of this study.

Our analyses show that, although each ascidian species has its own peculiar evolutionary rate, the nucleotide substitution rate is quite homogeneous at intraspecies level, thus allowing the easy recognition of extraordinary cases, such as those of our *B. schlosseri* specimens. Homogeneous values in the nucleotide substitution rate are observed even at congeneric level, with the lowest congeneric rates found in the comparisons between cryptic and morphologically very similar species. Noteworthy, the congeneric d*N* values are up to two orders of magnitude higher than those found at intraspecies level, highlighting the presence of a wide gap between intraspecies and congeneric evolutionary rates. The existence of this gap is again crucial to try to elucidate the biological meaning of extraordinary cases of intraspecies divergence, such as those found in the *B. schlosseri* Italian specimens. Indeed, the nucleotide substitution rate found in the Bs_Ita pairs is almost in between the rates observed at intraspecies and at congeneric level, but yet consistently lower than those found in cryptic (*C. intestinalis* sp.A–sp.B) and in morphologically very similar (*Botrylloides leachii*–*Botrylloides nigrum*) species pairs. These data suggest that our *B. schlosseri* specimens are the product of ongoing speciation events*.* Strikingly, the intraspecies divergence of the Bs_Ita pairs is easily detectable by whole-mitogenome nucleotide substitution analyses but indiscernible by the *cox1* barcode-fragment. Indeed, in the *cox1* phylogenetic tree, our four specimens fall within the same clade, whose internal relationships are however completely unresolved. This observation points to a higher resolving power of the whole mitogenome compared with *cox1*, especially in case of subtle speciation events. To further verify this hypothesis, it will be interesting to sequence and carry out comparative analyses on the entire mitogenome of specimens belonging to each of the different *B. schlosseri* clades/cryptic species identified by Bock et al. (2012): we can envisage that these mitogenomes will exhibit highly divergent sequences and d*N* values comparable with those found in congeneric comparisons.

We need to stress that the high resolving power at short phylogenetic distances of our whole-mitogenome comparative approach, coupled with the easy mitogenome sequencing through NGS technology, will be helpful for the investigation of speciation events and close phylogenetic relationships especially in taxa representing a challenge for morphological studies. Among ascidians, Botryllinae is one of these problematical taxa. Indeed, the debated Botryllinae classification has been revised several times (Saito and Okuyama 2003; Brunetti 2009). Moreover, inconsistencies in the morphological description of *B. schlosseri* have already suggested that more than one species is currently classified as *B. schlosseri* (Boyd et al. 1990).

In conclusion, our study underlines how comprehensive mt comparative analyses of the nucleotide substitution rate, calculated separately for the different functional mitogenomic regions, is able to solve debatable taxonomic questions at short phylogenetic distances, such as the existence of subtle ongoing speciation events. However, to reach this goal, comparative analyses need to be performed on an appropriate number and type of conspecific and congeneric comparisons. Thus, the mitogenomic comparative approach is valid at low taxonomic level even in absence of significant differences in genome structure, such as those observed in the *C. intestinalis* cryptic species (Iannelli, Pesole, et al. 2007).

## Supplementary Material

Supplementary figures S1–S3 and tables S1 and S2 are available at *Genome Biology and Evolution* online (http://www.gbe.oxfordjournals.org/).

Supplementary Data
