# Supplementary material for: Ascidian Mitogenomics: Comparison of Evolutionary Rates in Closely Related Taxa Provides Evidence of Ongoing Speciation Events
Source: Genome Biol Evol. 2014 Feb 25;6(3):591–605. doi: 10.1093/gbe/evu041 (PMC3971592; doi:10.1093/gbe/evu041)
Supplement: Supplementary Data [file supp_6_3_591__index.html]

Ascidian mitogenomics: comparison of evolutionary rates in closely related taxa provides evidence of ongoing speciation events — Ascidian Mitogenomics: Comparison of Evolutionary Rates in Closely Related Taxa Provides Evidence of Ongoing Speciation Events — Supplementary Data 

# Ascidian Mitogenomics: Comparison of Evolutionary Rates in Closely Related Taxa Provides Evidence of Ongoing Speciation Events

## Supplementary Data

files

**Files in this Data Supplement:**

- Supplementary Data - pdf file
- Supplementary Data - pdf file
- Supplementary Data - pdf file
- Supplementary Data - xls file
- Supplementary Data - xls file
