# Supplementary figures and images for: Ascidian Mitogenomics: Comparison of Evolutionary Rates in Closely Related Taxa Provides Evidence of Ongoing Speciation Events
Source: Genome Biol Evol. 2014 Feb 25;6(3):591–605. doi: 10.1093/gbe/evu041 (PMC3971592; doi:10.1093/gbe/evu041)

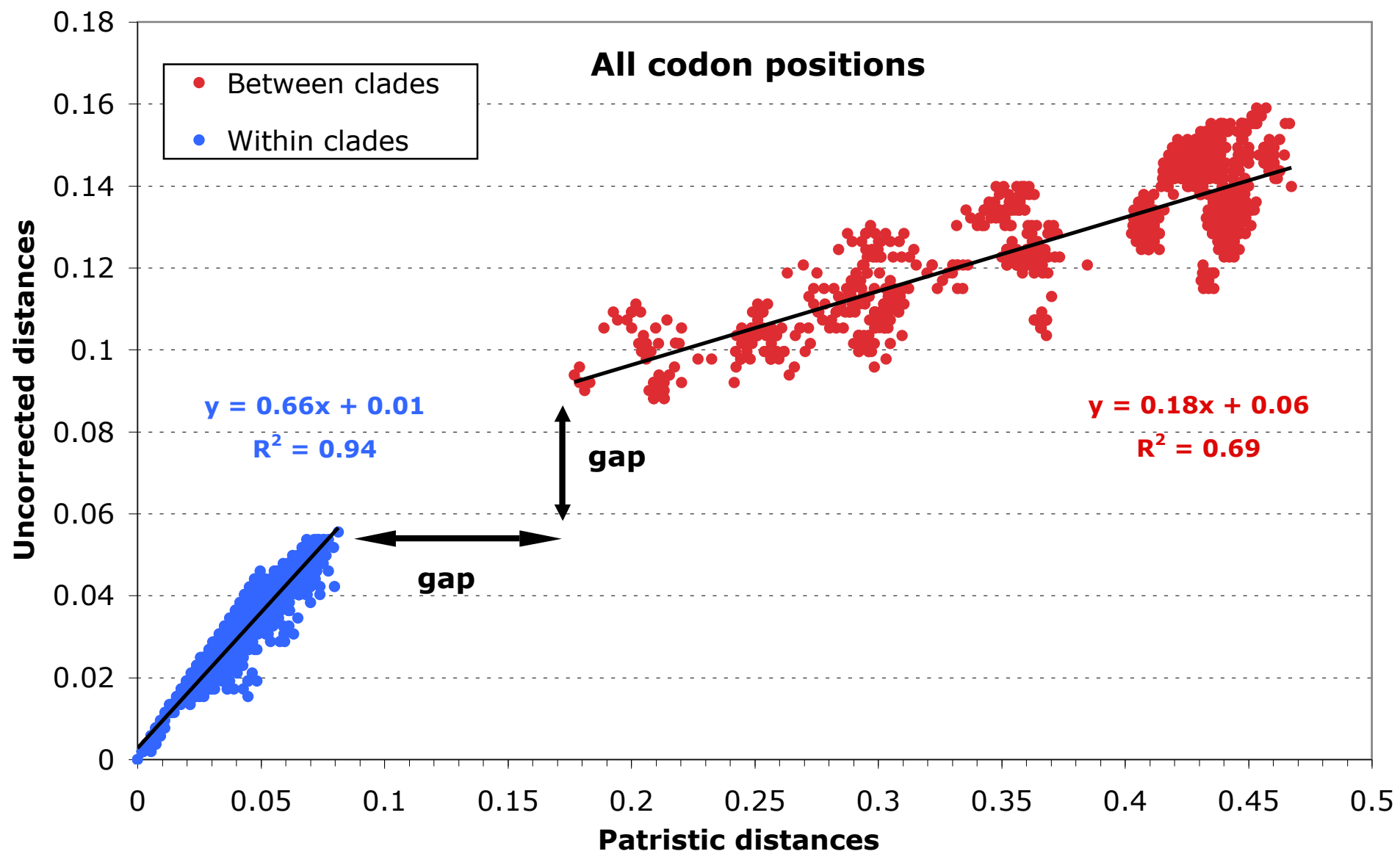

Supplement: Supplementary Data [file supp_evu041_Figure_S1.pdf]

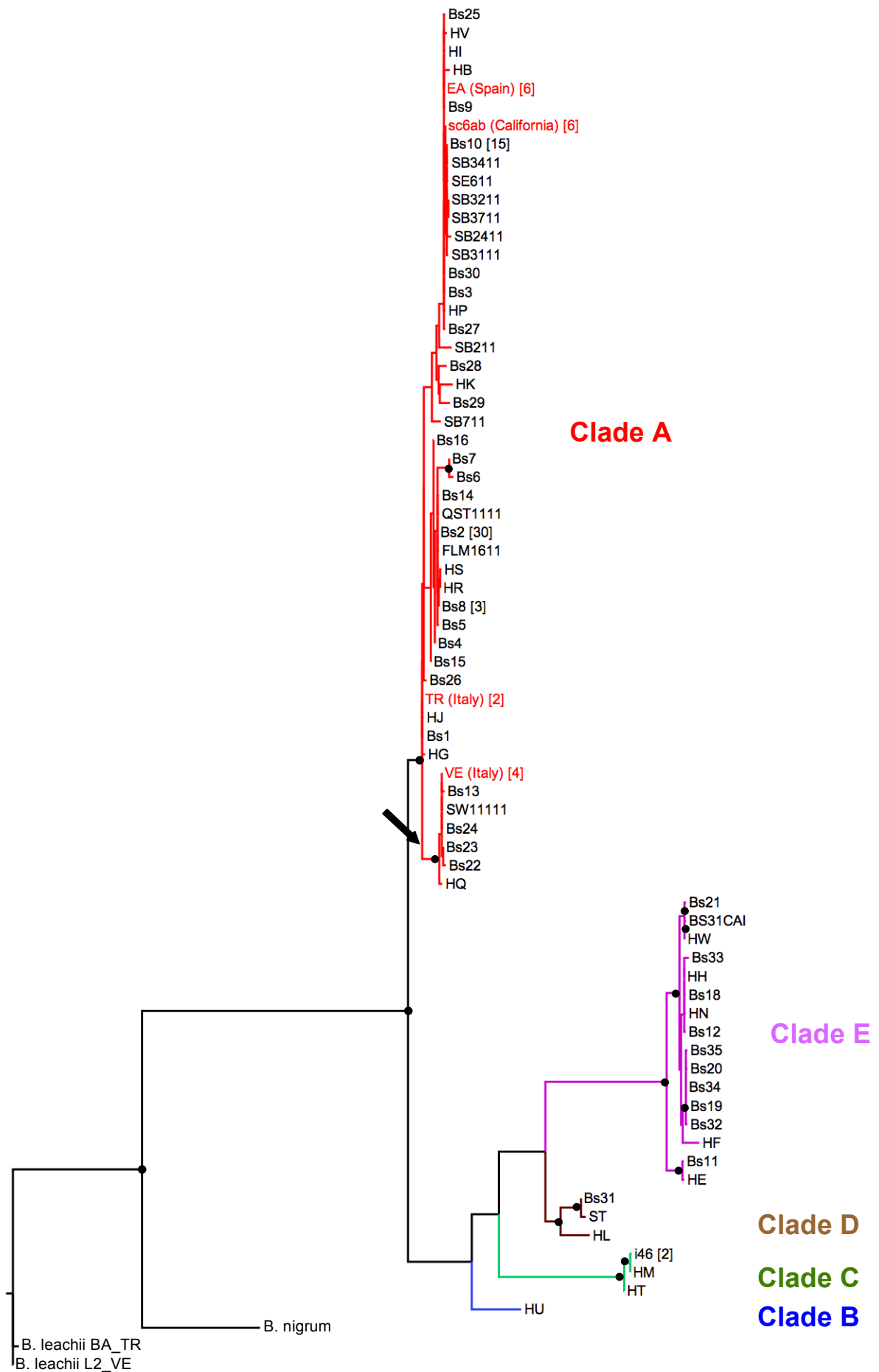

Supplement: Supplementary Data [file supp_evu041_Figure_S2.pdf]
