# Supplementary material for: Ascidian Mitogenomics: Comparison of Evolutionary Rates in Closely Related Taxa Provides Evidence of Ongoing Speciation Events
Source: Genome Biol Evol. 2014 Feb 25;6(3):591–605. doi: 10.1093/gbe/evu041 (PMC3971592; doi:10.1093/gbe/evu041)
Supplement: Supplementary Data [file supp_evu041_fig_S3.pdf]

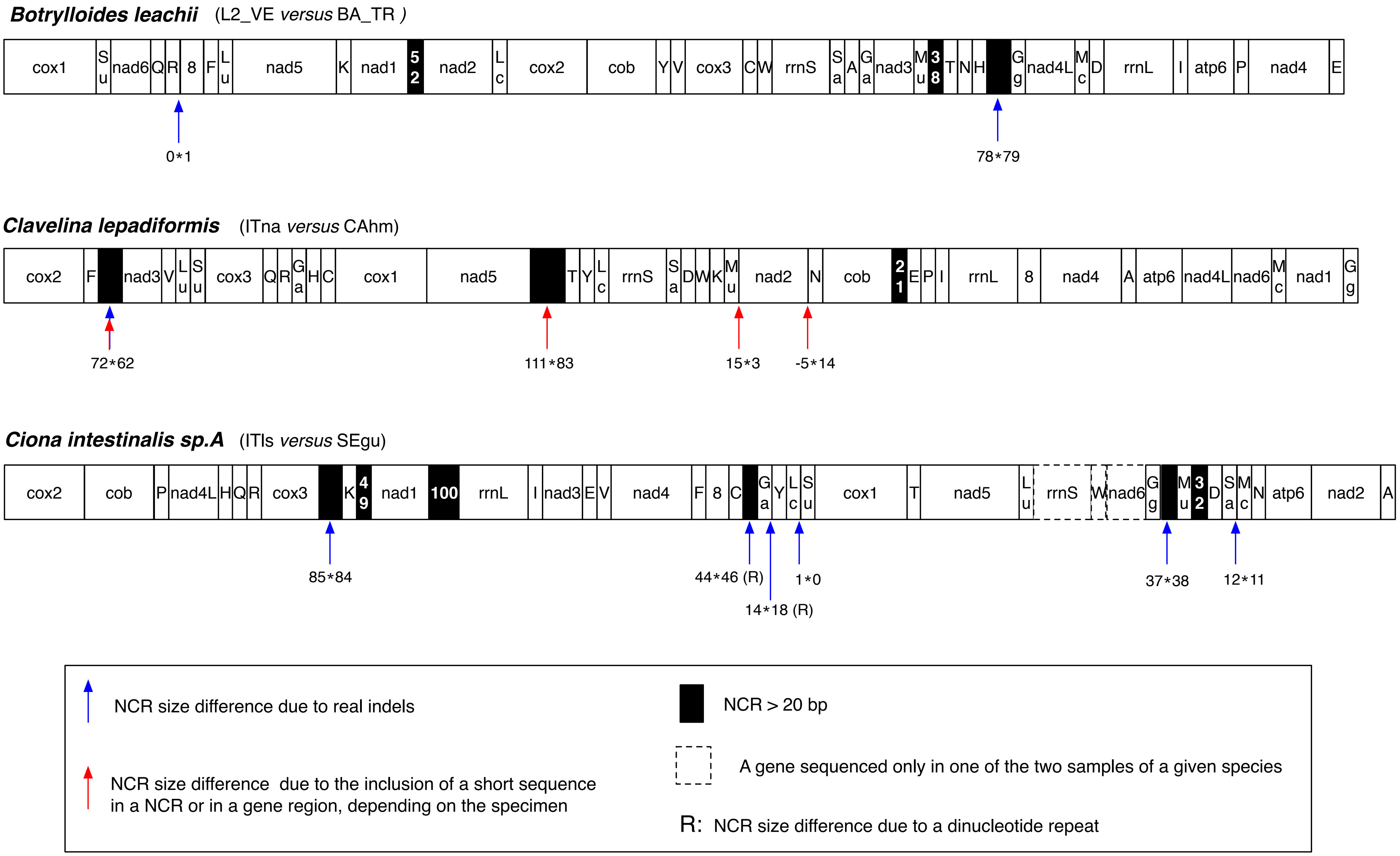

Botrylloides leachii (L2_VE versus BA_TR)
cox1 S u nad6 Q R 8 F L u nad5 K nad1 5 2 nad2 L c cox2 cob Y V cox3 C W rrnS S a A G a nad3 M u 3 8 T N H G g nad4L M c D rrnL I atp6 P nad4 E
0*1
78*79
Clavelina lepadiformis (ITna versus CAhm)
cox2 F nad3 V L u S u cox3 Q R G a H C cox1 nad5 T Y L c rrnS S a D W K M u nad2 N cob 2 1 E P I rrnL 8 nad4 A atp6 nad4L nad6 M c nad1 G g
72*62
111*83
15*3
-5*14
Ciona intestinalis sp.A (ITIs versus SEgu)
cox2 cob P nad4L H Q R cox3 K 4 9 nad1 100 rrnL I nad3 E V nad4 F 8 C G a Y L c S u cox1 T nad5 L u rrnS W nad6 G g M u 3 2 D S a M c N atp6 nad2 A
85*84
44*46 (R)
14*18 (R)
1*0
37*38
12*11
NCR size difference due to real indels
NCR size difference due to the inclusion of a short sequence in a NCR or in a gene region, depending on the specimen
NCR > 20 bp
A gene sequenced only in one of the two samples of a given species
R: NCR size difference due to a dinucleotide repeat
